# Machine-Learning Classification of Pulse Waveform Quality

**DOI:** 10.3390/s22228607

**Published:** 2022-11-08

**Authors:** Te Ouyoung, Wan-Ling Weng, Ting-Yu Hu, Chia-Chien Lee, Li-Wei Wu, Hsin Hsiu

**Affiliations:** 1 Division of Family Medicine, Department of Family and Community Medicine, Tri-Service General Hospital, National Defense Medical Center, Taipei 114, Taiwan; 2 School of Medicine, National Defense Medical Center, Taipei 114, Taiwan; 3 Health Management Center, Department of Family and Community Medicine, Tri-Service General Hospital, National Defense Medical Center, Taipei 114, Taiwan; 4 Graduate Institute of Biomedical Engineering, National Taiwan University of Science and Technology, Taipei 10607, Taiwan

**Keywords:** waveform quality, pulse, spectral analysis, machine learning, contacting pressure

## Abstract

Pulse measurements made using wearable devices can aid the monitoring of human physiological condition. Accurate estimation of waveforms is often difficult for nonexperts; motion artifacts may occur during tonometry measurements when the skin–sensor contact pressure is insufficient. An alternative approach is to extract only high-quality pulses for use in index calculations. The present study aimed to determine the effectiveness of using machine-learning analysis in discriminating between high-quality and low-quality pulse waveforms induced by applying different contact pressures. Radial blood pressure waveform (BPW) signals were measured noninvasively in healthy young subjects using a strain-gauge transducer. One-minute-long trains of pulse data were measured when applying the appropriate contact pressure (67.80 ± 1.55 mmHg) and a higher contact pressure (151.80 ± 3.19 mmHg). Eight machine-learning algorithms were employed to evaluate the following 40 harmonic pulse indices: amplitude proportions and their coefficients of variation and phase angles and their standard deviations. Significant differences were noted in BPW indices between applying appropriate and higher skin–surface contact pressures. The present appropriate contact pressure could not only provide a suitable holding force for the wearable device but also helped to maintain the physiological stability of the underlying tissues. Machine-learning analysis provides an effective method for distinguishing between the high-quality and low-quality pulses with excellent discrimination performance (leave-one-subject-out test: random-forest AUC = 0.96). This approach will aid the development of an automatic screening method for waveform quality and thereby improve the noninvasive acquisition reliability. Other possible interfering factors in practical applications can also be systematically studied using a similar procedure.

## 1. Introduction

Wearable systems monitoring health status provide significant benefits in healthcare applications [1,2]. The arterial pulse is generated by the propelling force of the heartbeat and is transmitted along the artery to the peripheral vascular beds; the pulse waveform determined by interactions between the heart pumping and the arterial system can provide information about the stiffness of the arterial wall and the blood flow [3]. Since the pulse waveform can easily be measured noninvasively by using wearable devices, pulse measurements and analyses have been considered useful for monitoring the physiological or pathological condition of the human body. 

Several diseases can induce changes in arterial stiffness and thereby affect the amplitude and speed of arterial pulse-wave transmission and thus change the arterial pulse waveform. Therefore, several indices have been used to capture changes in the pulse waveform related to disease-induced physiological changes [4]. For example, pulse analysis has been suggested as having tremendous potential in detecting conditions, such as cardiac arrhythmia and hypertension, that can lead to more-serious cardiovascular diseases [5]. The pulse-wave velocity and the augmentation index are widely used indices calculated from the time-domain pulse waveform. Aging, hypertension [6], hypercholesterolemia [7], coronary artery disease [8], and peripheral cardiovascular disease [4] have been found to influence arterial properties and hence change certain time-domain indices.

The pulse waveform can also be described by frequency-domain indices, with specific patterns having been noted in pulse indices in patients with cardiovascular diseases, such as cerebrovascular disease [9] and stroke [10], as well as in noncardiovascular diseases, such as metabolic syndrome [11], dementia [12], polycystic-ovary syndrome [13], breast cancer [14], and frozen shoulder [15]. These findings indicate that analyses of the pulse waveform can be useful for monitoring changes in the vascular properties induced by many kinds of diseases.

Both mechanical and optical devices are currently used to detect arterial pulses on the skin surface [16]. Pulse oximetry relies on measurements of transmittance or reflectance photoplethysmography (PPG) signals produced by variations in the quantity of arterial blood associated with the periodic contractions and relaxations of the heart [1]. Applanation tonometry can be used to measure the blood pressure waveform (BPW) from the radial artery [5]. Contactless monitoring methods have also been used to acquire the pulse waveform, such as an ultrawideband Doppler radar system for measuring the arterial blood flow condition and a millimeter-wave radar system for characterizing the pulse motion near the radial artery without touching the skin [5]. These systems may avoid the sensor-placing problem associated with tonometer-based approaches [17].

Wearable devices can be used for the low-cost, noninvasive, and continuous monitoring of the pulse waveform. However, in practical applications, it is often difficult to accurately estimate waveform indices, especially for nonexperts. Such artifacts may give rise to erroneous interpretations and false alarms of the pulse indices in clinical physiological measurements [18]. For example, it is easy for BPW and PPG signals to be contaminated with noise artifacts induced by the movements of the subject or the measurement apparatus [19]. Moreover, considerable interindividual and sensor-location variabilities are often present [18]. For optical PPG measurements, the ambient lighting, wearing different types of nail polish, and poor blood flow in the peripheral tissues can severely affect the quality and accuracy of the acquired waveforms [19]. Moreover, since BPW tonometry yields pressure signals, motion artifacts are often difficult to suppress or eliminate, and the mechanical displacements of the probe may also induce motion artifacts. Such motion artifacts are often difficult to filter out because their spectrum may overlap with that of the pulse signal, making the contaminated pulses beyond reparation. Therefore, ensuring a stable measurement condition is critical to accurate and reliable measurements [20].

Facing these interferences that may especially occur outside a laboratory environment, an alternative approach is to assess the signal quality of each pulse waveform and then extract only high-quality pulses for use in further index calculations. The development of algorithms capable of removing—or at least identifying—artifacts in the recorded pulse data is therefore of critical importance [20]. Machine-learning analysis has been widely used to construct effective classifier algorithms for distinguishing data that are not linearly separable. Such techniques are already widely employed to estimate cardiovascular indices or to predict the cardiovascular health condition, such as in diagnosing arterial hypertension [21,22], and for estimating the aortic pulse-wave velocity, stroke volume, and blood pressure (BP) [23]. The often subtle and complex nature of changes in the pulse waveform make machine-learning analysis particularly suitable for developing algorithms that can identify the quality of pulse waveforms. 

When wearable sensors are attached to the human body to measure pulse signals, relative displacement between the sensor and the skin surface can occur. Taking PPG measurements as an example, the relative displacement may change the location and contacting condition of the optical sensor and hence change the illumination or receiving location. For tonometry measurements, motion artifacts may occur when the contact pressure between the sensor and the skin is insufficient. Numerous factors can distort the pulse waveform, and their interfering effects can differ. It is therefore necessary to consider the interfering effect corresponding to each factor separately in order to obtain a clearer understanding and also to develop the corresponding distortion-detection algorithm for the effects on the pulse waveform. Since the heartbeat-driven pulse signal is quasiperiodic, beat-to-beat artifact detection should be provided in the algorithm. Beat-to-beat analysis can not only help to increase the amount of acquired data (which is beneficial to machine-learning analysis) but also provide more-detailed information about the waveform quality of the acquired pulse data sequence.

The aims of the present study were (1) to determine and compare the effects on the tonometry pulse waveform (using strain-gauge transducers to measure the radial arterial BPW) of applying appropriate and higher contact pressures and (2) to determine the effectiveness of using machine-learning analysis in automatically discriminating between high-quality and low-quality pulse waveforms induced by applying different contact pressures. Since changes in the pulse waveforms are subtle and often difficult to detect, two strategies were adopted in order to enhance the discriminating ability: (1) frequency-domain analysis to minimize the possible distortion effects on time-domain pulse waveform caused by small perturbations induced by motion artifacts and other factors and (2) machine-learning techniques using frequency-domain waveform indices as features to detect the induced differences in the spectral patterns. The obtained knowledge may be useful in the development of a wearable device for automatically detecting the abnormal pulse waveforms induced by different contact pressures.

## 2. Materials and Methods

Details of the present experimental setup and the signal processing methods are available elsewhere [11,12,15] and in the Supplementary Materials. The subjects were all graduate students of the National Taiwan University of Science and Technology (for details, see Table 1). Written informed consent was obtained from each study participant (approved by the Research Ethics Committee of Tri-Service General Hospital; TSGHIRB 2-108-05-161), and all experiments were performed in accordance with relevant guidelines and regulations. Subjects were excluded if they did not agree to participate in the study or were unable to cooperate with the research steps, such as due to their limbs trembling involuntarily.

### 2.1. Measurements

The study procedures were designed as follows:

(1) Perform measurements on healthy young subjects.
(2) Calculate the harmonic indices and compare the pulse waveforms between applying appropriate and higher contact pressures on the same subject.
(3) Extract the high-quality and low-quality pulses in measurements made using appropriate and higher contact pressures, respectively, according to the judgment of two experts.
(4) Calculate the harmonic indices and compare the results between high-quality and low-quality pulse waveforms.
(5) Use the harmonic indices as features to classify high-quality and low-quality pulse waveforms using machine-learning analysis.

**Figure 1:**
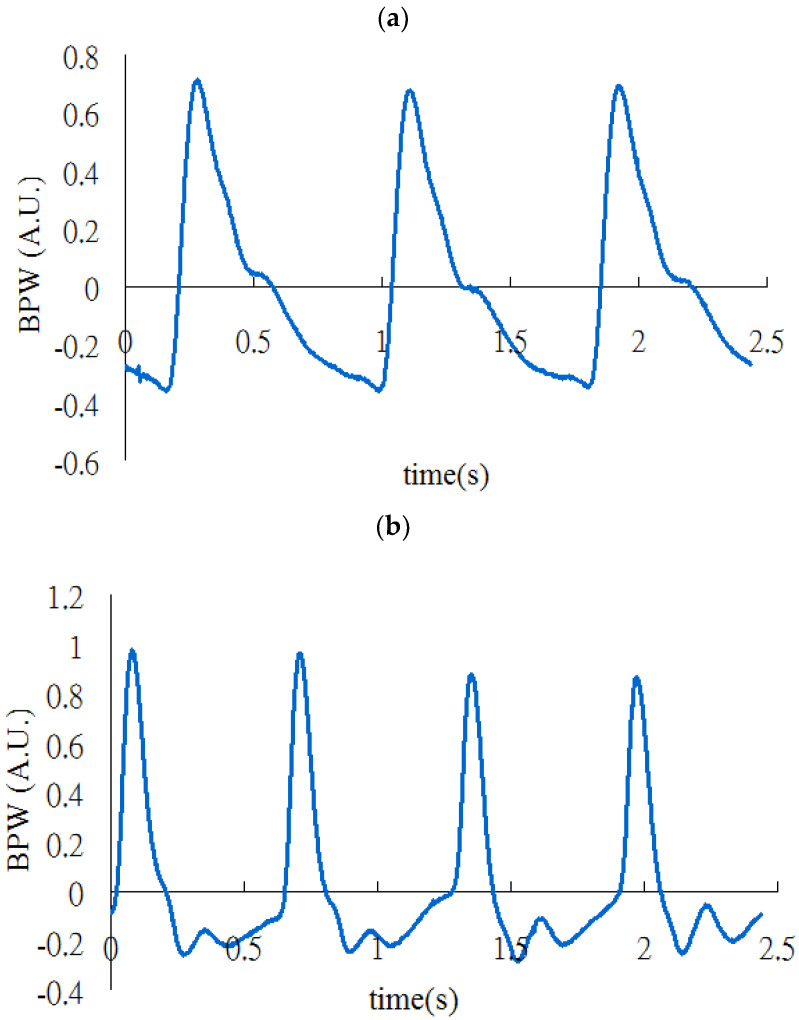
Typical measured pulse waveforms for (**a**) Data A (high quality) and (**b**) Data B (low quality).

The BPW signal was noninvasively measured in the subjects (typical waveforms are shown in Figure 1). The BPW signal was acquired and sampled at 1024 Hz by a pressure transducer (KFG-2-120-D1-11, Kyowa, Hong Kong, China) held onto the skin surface by an elastic belt above the radial artery 2 cm from the left wrist. Before the measurement, the heart rate (HR), brachial systolic BP, and diastolic BP were measured using a sphygmomanometer (MG150f, Rossmax, Taipei, Taiwan) [12,15].

Before the measurements, the subjects sat rested in a chair for 10 min. We first measured the circumference of the wrist (*L*) and then chose the length of the elastic belt as *L* to provide appropriate contact pressure between the sensor and the skin surface. We then shortened the elastic belt in steps of 0.5 mm to gradually increase the contact pressure in order to determine the belt length required for the higher contact pressure. The body structure, such as the bone size and weight, can be different between genders. In the present study, the belt lengths that resulted in a higher contact pressure were chosen as *L* minus 2.0 mm and *L* minus 1.5 mm for male and female subjects, respectively. The appropriate and higher contact pressures were measured to be 67.80 ± 1.55 mmHg (mean ± standard deviation) and 151.80 ± 3.19 mmHg, respectively.

For each subject, we compared the waveforms between appropriate and higher contact pressures according to the following procedure: 1 min of pulse data measured by applying the appropriate contact pressure, 3 min of rest, shortening the belt, followed by another 1 min of measurements when applying the higher contact pressure.

To prepare the database for the beat-to-beat comparisons, manual annotations for all records were performed by two experts. By observing the BPW pulse shape, the experts could classify each BPW pulse as being of either high or low quality. The high-quality pulses (Data A) were selected from the measurements when applying the appropriate contact pressure, whereas the low-quality pulses (Data B) were selected from measurements when applying the higher contact pressure. High-quality pulses constituted 98.0% of the measurements made when applying the appropriate contact pressure, whereas low-quality pulses constituted 78.2% of the measurements made when applying the higher contact pressure. The performance of the machine-learning algorithms in classifying the high-quality and low-quality pulses could then be estimated.

### 2.2. Analysis

There are two parts in the present analysis procedure: signal processing and information processing. In signal processing, frequency-domain analysis was performed to derive the following 40 BPW harmonic indices for *n* = 1–10: amplitude proportion (*C_n_*), coefficient of variation of *C_n_* (*CV_n_*), phase angle (*P_n_*), and standard deviation of *P_n_* (*P_n_*_*SD*). These 40 indices for each pulse (*n* = 1–10) were then used as the features of the ML analysis: *C_n_*, *CV_n_*, *P_n_*, and *P_n_*_*SD*.

**Figure 2:**
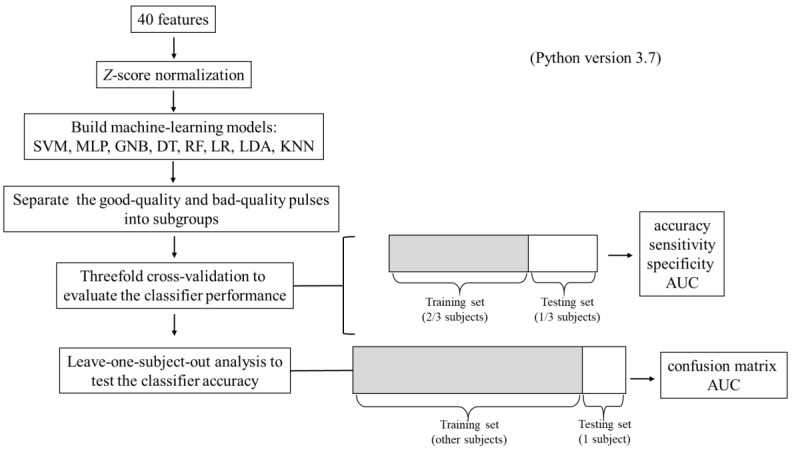
Flowchart of information processing in the study. Forty indices (*C_n_*, *P_n_*, *CV_n_*, and *P_n_*_*SD*; *n* = 1–10) were calculated, which were used as the features for the information processing. Threefold cross-validation was used to evaluate the model performance, and then leave-one-subject-out analysis was used to test the model accuracy.

In information processing, ML analysis was used to discriminate between groups (details are provided in the Supplementary Materials; processing procedure is shown in Figure 2). Eight supervised methods were used for the binary classification of the data, which were support vector machine (SVM), multilayer perceptron (MLP), Gaussian Naïve Bayes (GNB), decision tree (DT), random forest (RF), logistic regression (LR), linear discriminant analysis (LDA), and K-nearest neighbor (KNN). Threefold cross-validation was used in the model training process. The classification ability between the high- and low-quality pulses of the proposed classification models was tested by a leave-one-subject-out test. The confusion matrix was determined, and the accuracy, sensitivity, specificity, and AUC of ROC were calculated to evaluate the classifier performance.

## 3. Results

**Figure 3:**
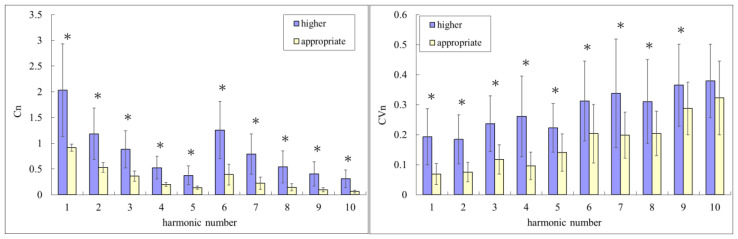

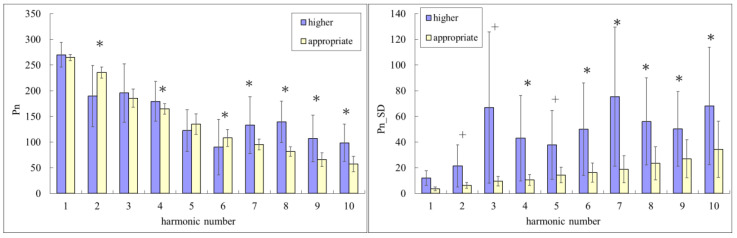
Comparisons of harmonic indices between applying the appropriate and higher contact pressures: *C_n_*, *CV_n_*, *P_n_*, and *P_n_*_*SD*. Data are mean and standard-deviation values. *C*_6_–*C*_10_ values have been multiplied by 10 to make the differences clearer. “*” indicates *p* < 0.05; “+” indicates 0.05 < *p* < 0.1. All *C_n_* and *CV*_1_–*CV*_9_ were significantly larger when applying higher-than-appropriate contact pressure. Regarding phase-angle indices, there were significant differences in *P*_2_, *P*_4_, and *P*_6_–*P*_10_. Regarding *P_n_*_*SD* indices, *P*_2__*SD*–*P*_10__*SD* were either significantly (*p* < 0.05) or marginally (0.05 < *p* < 0.1) larger when applying a higher-than-appropriate contact pressure.

The characteristics of the study subjects are listed in Table 1. Figure 3 compares the beat-to-beat parameters of BPW signals between applying the appropriate and higher contact pressures. Regarding BPW indices, almost all of the *C_n_* and *CV_n_* indices were significantly larger when applying a higher-than-appropriate contact pressure. Regarding phase-angle indices, there were significant differences in *P*_2_, *P*_4_, and *P*_6_–*P*_10_. Almost all *P_n_*_*SD* indices were either significantly (*p* < 0.05) or marginally (0.05 < *p* < 0.1) larger when applying higher-than-appropriate contact pressure. 

**Figure 4:**
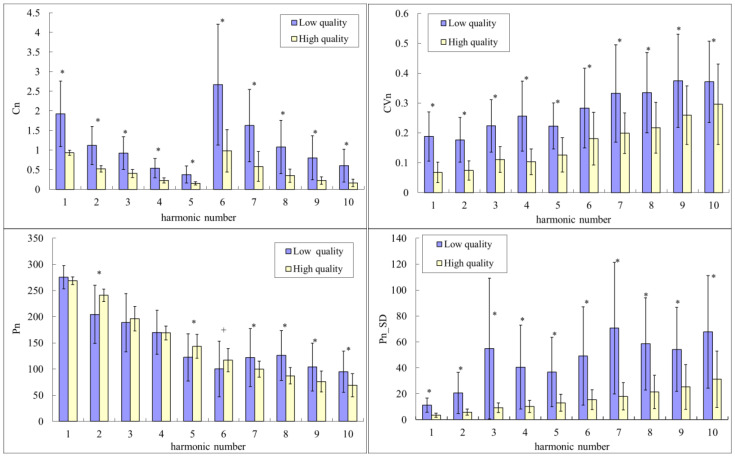
Comparisons of harmonic indices between Data A (high quality) and Data B (low quality): *C_n_*, *CV_n_*, *P_n_*, and *P_n_*_*SD*. Data are mean and standard-deviation values. *C*_6_–*C*_10_ values have been multiplied by 10 to make the differences clearer. “*” indicates *p* < 0.05. “+” indicates 0.05 < *p* < 0.1. *C_n_*, *CV_n_*, and *P_n_*_*SD* were all significantly larger for Data B than for Data A. Regarding phase-angle indices, there were significant differences in *P*_2_, *P*_5_, and *P*_7_–*P*_10_.

After selecting pulses according to the judgment of the experts, Figure 4 compares the beat-to-beat parameters of BPW signals between Data A (high quality) and Data B (low quality). Figure 4 indicates that there were significant differences in most BPW indices; for example, all *C_n_*, *CV_n_*, and *P_n_*_*SD* indices were significantly larger for Data B than for Data A. Regarding phase-angle indices, there were significant differences in *P*_2_, *P*_5_, and *P*_7_–*P*_10_.

The results of the eight machine-learning analysis methods are listed in Table 2 and Table 3. The threefold cross-validation analysis results listed in Table 2 indicate that all eight methods had AUCs of around 0.80 (range, 0.74–0.85). The AUC was largest for MLP and RF, 0.83 and 0.85, respectively. The mean accuracy, sensitivity, and specificity were 83.54%, 0.84, and 0.88, respectively, for MLP, and 85.36%, 0.88, and 0.85 for RF. 

The results of the leave-one-subject-out analysis listed in Table 3 indicate that all eight methods had AUCs of around 0.90 (range, 0.78–0.96). The AUC was largest for RF and KNN, at 0.96 and 0.93, respectively. The accuracy, sensitivity, and specificity were 88.33%, 80.00%, and 96.67%, respectively, for RF. These values indicate that, when using BPW pulse indices as features, RF analysis facilitates the ability to distinguish high-quality from low-quality pulse waveforms with high accuracy and specificity.

For the BPW indices, we performed hold-out analysis to further evaluate the model accuracy (using threefold cross validation during evaluation and splitting the training set and validation data as 8:2). For RF as listed in Table 4, the mean accuracy, sensitivity, specificity, and AUC were 84.25%, 0.74, 0.92, and 0.84, respectively, for the threefold cross validation and were 80.56%, 0.99, 0.68, and 0.84, respectively, for the hold-out test. Another method with high AUC in a hold-out test is the KNN; the accuracy, sensitivity, specificity, and AUC were 84.78%, 0.92, 0.80, and 0.86, respectively, for the hold-out test.

## 4. Discussion

The present study found significant differences in several BPW indices between applying appropriate and higher skin-surface contact pressures. By choosing high-quality and low-quality pulses from these two groups, the machine-learning analysis using frequency-domain pulse indices as features can aid the identification of the quality of pulse waveforms.

Several previous studies have investigated the use of waveform analysis to discriminate between contaminated and noncontaminated pulses, and most of them focused on PPG signals [18,19,20]. For example, the interfering effects of varying degrees of purposely induced motion artifacts in a laboratory environment have been studied, with an accuracy of 95.2% found for the test set, and the authors concluded that the algorithm could be deployed as a signal-quality-assessment algorithm for PPG signals [18]. Gold-standard PPG signals and PPG signals contaminated by purposely induced artifact noise from a variety of activities involving subject movements were compared in another study. Using waveform morphology analysis and a decision-tree classifier, an 83% accuracy was achieved in identifying high-quality pulses [19]. Fischer et al. used decision lists to discriminate between disturbed and undisturbed pulses, with their algorithm achieving an accuracy of 98.3% [20].

Compared with PPG signals, the BPW signals acquired around the radial artery travel a shorter distance to the main artery. This characteristic may facilitate the monitoring of the pulse propagation condition on the main artery and also help to reduce the interfering effects of the changes in the peripheral microcirculation at the extremity (which are often encountered in PPG measurements). However, for wearable-device designs, it can be more difficult to achieve stable pulse waveforms for BPW measurements, since the anatomic structure of the lower arm is more complex than that of the finger. Tonometric transducers exhibit excellent sensitivity in acquiring BPW signals, but they often need careful handling and exact positioning over the artery [16]. Therefore, although BPW measurements have some application advantages over PPG measurements, an algorithm for identifying the quality of pulse waveforms is urgently needed for BPW measurements.

Compared with the results in the above-described previous PPG studies, the present findings indicated similar performance in discriminating between high-quality and low-quality pulses. The threefold cross-validation results of the eight machine-learning methods indicated that MLP and RF had the highest AUCs, at 0.83 and 0.85, respectively. Meanwhile, in the leave-one-subject-out tests, RF and KNN had the highest AUCs, at 0.96 and 0.93, respectively. These values indicate the excellent performance of RF analysis in discriminating between high-quality and low-quality pulses. In practical wearable-device applications, recording for 0.5–2.0 min can be necessary to acquire sufficient numbers of pulses, and rapid judgments can be made using machine-learning analysis (taking around several seconds). This means that the user will not perceive a marked difference in the time required when using a wearable device.

When contact pressure is exerted on the skin surface, it can be transmitted through the tissue onto the blood vessel walls of the peripheral vascular beds. Applying a contact pressure to the skin surface may therefore induce several types of physiological effects in the peripheral blood circulation [24,25,26]. Intuitively, the prolonged compression of vascular soft tissues can impair the peripheral blood flow; for example, lying in a hospital bed generates prolonged skin-to-bed pressures of 55–95 mmHg, which can produce local capillary ischemia [26]. However, applying contact pressure to the skin surface can also cause pressure-induced vasodilation (PIV) that increases blood flow. 

Different degrees of vessel compression may induce different degrees of deformation and thus different vascular regulatory responses. A vessel-pressing model (VPM) has previously been proposed for describing and explaining the relationship between the different effects induced by applying different contact pressures around the normal BP [27,28,29]. Contact pressure around the normal BP can elicit a prominent PIV response and hence lead to improvement in the blood flow. However, increasing the applied contact pressure will result in the greater compression of the vessel, such that the blood flow can be further obstructed so as to make it more difficult to elicit the PIV response in the local vascular beds. One possible important mechanism underlying the vascular response when applying a contact pressure much higher than the normal BP is the local vessel dilation induced by decreased sympathetic nerve activity [27].

Furthermore, for skin-surface pulse measurements, it was found that applying the sensor with a gentle contact pressure may improve the detection capacities for both PPG [1,16] and wrist tonometry [5] measurements; such forces should be kept low to avoid pain and the blocking of the venous flow during long-term wearing [16]. There is a range of contact forces that yield optimal PPG signals and the highest PPG amplitudes [1], with insufficient contact pressure causing weak PPG signals and pressures that are too high blocking the blood circulation and deforming the PPG waveform [30]. For PPG measurements, contact pressures in the range of 8–12 kPa result in the highest PPG amplitudes, while those in the range of 4–40 kPa do not degrade the waveform quality [1]. The optimum pressure range for SpO_2_ detection was found to lie between 5 kPa and 15 kPa [30]. Tensions of up to 2 N produce no discomfort at the wrist and are found to be high enough to increase the PPG pulsatile output [16]. However, the optimal contact-pressure ranges for use in tonometry pulse measurements have been unclear.

The present findings indicated that the contact pressure can markedly affect the reliability of pulse-wave measurements and that the present method of machine-learning analysis can aid the ability to discriminate between high-quality and low-quality pulses. The present studied condition of using different applied contact pressures is a commonly encountered factor in practical applications, which means that the present findings have meaning in the quality control of data acquired using wearable devices for measuring the pulse waveform. Furthermore, several previous studies used the pulse amplitude as the indicator of the waveform quality [1,16,30]. The present study used frequency-domain analysis to provide an alternative description of the pulse waveform, which could help to evaluate the effects between different contact pressures in more detail. 

The present adopted mean value for the appropriate contact pressure (67.80 mmHg) was around the normal diastolic BP. According to the previous relevant studies discussed above, the used contact-pressure range was also around the normal BP; for example, 10 kPa corresponds to around 75 mmHg [1]. This illustrates that the effects on the measured pulse waveform can be partly attributed to the vessel deformation caused by the applied contact pressure. Similar to the conjecture of the VPM, if the contact pressure is around the BP, the more-abundant blood supply within the vessel may increase the resistivity against the contact pressure applied outside the vessel and hence provide a more-robust condition for pulse-waveform measurements. However, when the applied contact pressure is higher than the BP, the associated greater compression of the vessel may change the arterial pulse transmission condition and hence lead to distorted pulse waveforms. A similar condition was noted in one of our previous studies, which found that applying a contact pressure of around 60 mmHg resulted in an optimal correlation between radial tonometry and finger PPG pulse waveforms in terms of the amplitudes of their harmonic components [27]. This provides a method to reconstruct the arterial pulse waveform from the finger PPG signal, which has also been used to study the characteristics of the pulse waveform in subjects with metabolic syndrome and dementia [11,12,27]. 

## 5. Conclusions

When acquiring the heartbeat or pulse signals using wearable devices, the heartbeat interval of around 1 s makes it necessary to maintain a stable measurement condition for the duration required to accumulate sufficient pulses to fulfill the fundamental mathematical requirements of the analysis. For example, if 30 pulses are necessary for the analysis and the heart rate is 72 beats/s, the wearable device will need to record for about 30/72 × 60 = 25 s. Moreover, the wearable device needs to apply sufficient contact pressure to minimize the interfering effects of motion artifacts. Furthermore, to avoid the interference induced by different physiological conditions, it is also necessary to keep the physiological condition of the local blood supply stable while the wearable device is being applied. 

The present findings may be useful in practical applications of wearable devices. Ensuring that the skin-surface contact pressure remains within the appropriate range may satisfy both of the above-mentioned requirements: (1) providing a sufficient holding force for the wearable device and (2) helping to maintain the physiological stability for contacting the skin surface and its underlying tissues. We have further applied frequency-domain analysis and machine-learning analysis to develop an effective method for classifying the high-quality and low-quality pulses acquired by a wearable device, which yielded excellent discrimination performance (RF AUC = 0.96). This may aid the development of a method for the automatic screening of pulse-waveform quality and therefore help to improve the reliability of pulse-waveform measurements when using various types of wearable devices. Furthermore, similar to the present findings regarding the effects of applying different contact pressures, the effects of other possible interfering factors in practical wearable applications could also be systematically studied using the procedures similar to those employed in the present study. To enhance the discriminating effectiveness, adding more eigenvalues can be an important issue in further studies.

## Figures and Tables

**Table 1:** Characteristics of the subjects.

Subject number	60
Gender (male/female)	33/27
Age (years)	23.6 ± 0.8
BMI	20.03 ± 2.05
HR (beats/min)	69.57 ± 10.73
SBP (mmHg)	118.80 ± 9.49
DBP (mmHg)	75.21 ± 6.93

**Table 2:** Results of the machine-learning analyses comparing BPW indices between Data A (high quality) and Data B (low quality). Training and validation results are presented for the threefold cross-validation. For BPW indices, there were 1337 and 1026 pulses for Data A and Data B, respectively.

Accuracy (%)	SVM	MLP	GNB	DT	RF	LR	LDA	KNN
1	74.97	79.16	72.30	76.62	79.42	76.37	80.18	78.14
2	79.95	85.03	84.26	81.60	88.07	82.99	86.17	79.31
3	80.84	86.42	69.42	76.40	88.58	83.12	78.81	86.17
Average	78.59	83.54	75.33	78.21	85.36	80.83	81.72	81.21
Sensitivity	SVM	MLP	GNB	DT	RF	LR	LDA	KNN
1	0.70	0.74	0.70	0.77	0.76	0.71	0.75	0.77
2	0.74	0.82	0.79	0.79	0.84	0.78	0.83	0.78
3	0.81	0.96	0.76	0.80	0.99	0.89	0.78	0.91
Average	0.75	0.84	0.75	0.79	0.86	0.79	0.79	0.82
Specificity	SVM	MLP	GNB	DT	RF	LR	LDA	KNN
1	0.92	0.94	0.77	0.76	0.88	0.93	0.96	0.80
2	0.98	0.91	0.97	0.87	0.97	0.97	0.93	0.82
3	0.80	0.78	0.63	0.72	0.80	0.77	0.79	0.81
Average	0.90	0.88	0.79	0.78	0.88	0.89	0.89	0.81
AUC	SVM	MLP	GNB	DT	RF	LR	LDA	KNN
1	0.72	0.76	0.70	0.76	0.77	0.73	0.77	0.77
2	0.77	0.84	0.82	0.80	0.87	0.81	0.85	0.78
3	0.80	0.88	0.70	0.76	0.90	0.84	0.78	0.86
Average	0.76	0.83	0.74	0.77	0.85	0.79	0.80	0.80

**Table 3:** Results of the leave-one-subject-out analysis for discriminating BPW indices between Data A (high quality) and Data B (low quality). “1” indicates high-quality pulses, and “0” indicates low-quality pulses. TP, true positive; FN, false negative; TN, true negative; FP, false positive. Accuracy = (TP + TN)/(TP + FN + TN + FP); sensitivity = TP/(TP + FN); specificity = N/(FP + TN).

	SVM	MLP	GNB	DT	RF	LR	LDA	KNN
TP	18	22	20	20	24	21	24	22
FN	12	8	10	10	6	9	6	8
TN	30	29	26	28	29	28	27	28
FP	0	1	4	2	1	2	3	2
Sensitivity (%)	60.00	73.33	66.67	66.67	80.00	70.00	80.00	73.33
Specificity (%)	100.00	96.67	86.67	93.33	96.67	93.33	90.00	93.33
Accuracy (%)	80.00	85.00	76.67	80.00	88.33	81.67	85.00	83.33
AUC of ROC	0.83	0.89	0.78	0.90	0.96	0.89	0.90	0.93

**Table 4:** Hold-out analysis results for comparisons of BPW indices between Data A and Data B.

Accuracy (%)	SVM	MLP	GNB	DT	RF	LR	LDA	KNN
1	88.96	90.18	85.89	80.83	95.25	94.33	89.42	89.26
2	78.08	77.29	78.23	71.77	79.18	77.29	82.49	71.13
3	75.85	79.38	70.30	78.00	78.31	77.69	76.46	78.15
Average	80.96	82.28	78.14	76.87	84.25	83.1	82.79	79.51
Hold-out test	77.05	71.43	58.08	73.07	80.56	70.49	79.39	84.78
Sensitivity	SVM	MLP	GNB	DT	RF	LR	LDA	KNN
1	0.76	0.92	0.76	0.77	0.99	0.97	0.77	0.97
2	0.58	0.59	0.62	0.56	0.64	0.63	0.66	0.59
3	0.46	0.60	0.53	0.69	0.60	0.49	0.53	0.58
Average	0.60	0.70	0.64	0.67	0.74	0.7	0.65	0.71
Hold-out test	0.78	0.82	0.78	0.96	0.99	0.77	0.75	0.92
Specificity	SVM	MLP	GNB	DT	RF	LR	LDA	KNN
1	0.98	0.89	0.93	0.84	0.92	0.92	0.98	0.84
2	0.98	0.95	0.94	0.87	0.94	0.91	0.98	0.82
3	0.97	0.93	0.83	0.84	0.91	0.98	0.93	0.92
Average	0.98	0.92	0.90	0.85	0.92	0.94	0.96	0.86
Hold-out test	0.76	0.64	0.45	0.58	0.68	0.66	0.82	0.80
AUC	SVM	MLP	GNB	DT	RF	LR	LDA	KNN
1	0.87	0.90	0.85	0.80	0.96	0.95	0.88	0.90
2	0.78	0.77	0.78	0.71	0.79	0.77	0.82	0.71
3	0.72	0.77	0.68	0.77	0.76	0.73	0.73	0.75
Average	0.79	0.81	0.77	0.76	0.84	0.82	0.81	0.79
Hold-out test	0.77	0.73	0.61	0.77	0.84	0.72	0.79	0.86

## Data Availability

The data presented in this study are available on request from the corresponding author. The data are not publicly available due to ethical concern.

## Supplementary Materials

The following supporting information can be downloaded at: https://www.mdpi.com/article/10.3390/s22228607/s1.
