# Supplementary material for: Machine-Learning Classification of Pulse Waveform Quality
Source: Sensors (Basel). 2022 Nov 8;22(22):8607. doi: 10.3390/s22228607 (PMC9698948; doi:10.3390/s22228607)
Supplement: Supplementary file 1 [file sensors-22-08607-s001.zip › sensors-1996782-supplementary.pdf]

Analysis

The present analysis procedure included signal processing and information processing:

- signal processing (implemented by MATLAB)

When determining the pulse waveforms, the signals were filtered by a digital 11th-order high-pass Chebyshev filter with a cut-off frequency of 0.01 Hz to eliminate the baseline drift and to provide a steeper transition region to improve the filtering effects on lower-frequency interference (such as motion artifact). Frequency-domain analysis was applied to derive the following four harmonic indices from the measured BPW signal: amplitude proportion (C_n), coefficient of variation of C_n (CV_n), phase angle (P_n), and standard deviation of P_n (P_n_SD).

Each individual pulse (between foot points) can be represented by the following finite series [12, 15]:

$$x(t) = \frac{A_0}{2} + \{\sum_{n=1}^{k/2} A_n \cos n\ \omega\ t_s + \sum_{n=1}^{k/2} B_n \sin n\ wt_s\}$$

The Fourier coefficients (A_n and B_n) of the pulse can be calculated as

$$A_n = \frac{2}{k}\sum_{s=0}^{k} x_s \cos n\ \omega\ t_s \ \ (\ for\ n = 0,1,...,\frac{k}{2}\ )$$

$$B_n = \frac{2}{k}\sum_{s=0}^{k} x_s \sin n\ \omega\ t_s \ \ (\ for\ n = 0,1,...,\frac{k}{2}\ )$$

where ω is the angular frequency and t_s is the sampling time interval.

The amplitude (Amp_n; n: the harmonic number) and phase angle (P_n) of each harmonic of the pulse harmonic spectrum can then be calculated as $Amp_n = \sqrt{A_n^2 + B_n^2}$ and $P_n = \arctan(B_n / A_n)$. The amplitude proportions (C_n values) for each pulse were calculated as $Amp_n / Amp_0 \times 100\%$, for n = 1–10. CV_n was then calculated as the coefficient of variations

(CV) of C_n, and P_n_SD was calculated as the standard deviation (SD) of P_n.

Signal processing was performed with MATLAB (MathWorks). The differences were tested with two-tailed t-test and were considered significant when $p<0.05$; all p-values were two-sided hypotheses.

■ information processing (implemented by Python version 3.7)

For information processing, the features of pulse signals were collected from the results of the signal-processing stage described above, to yield 40 indices for each pulse: C_n, CV_n, P_n, and P_n_SD values for n = 1–10. Before being input to the machine-learning analysis as features, CV_n and P_n_SD were recalculated for each pulse and the following 14 pulses. Each feature was scaled by Z-score normalization to eliminate the effects of the variations in the ranges of different indices.

Python (version 3.7) was used as the analysis tool in the information processing. Scikit-learn preprocessing package was used in the normalization. Eight supervised methods, including support vector machine (SVM), multilayer perceptron (MLP), Gaussian Naïve Bayes (GNB), decision tree (DT), random forest (RF), logistic regression (LR), linear discriminant analysis (LDA), and K-nearest neighbor (KNN) were used for the binary classification of the data. Details of the parameter settings for the eight methods are described below.

Parameters of the machine-learning models.

machine-learning methods	model parameters
SVM<br>(support vector machine)	C=1; kernel: rbf; gamma: auto; tol= 0.0001;<br>max_iter=-1; class_weight: none
MLP<br>(multilayer perception)	hidden_layer_sizes=100; solver: adam; alpha=0.0001;<br>batch_size: auto; max_iter=200; learning_rate_int=0.001
GNB<br>(Gaussian Naive Bayes)	Priors: none
DT<br>(decision tree)	Criterion: gini; Splitter: best; max_depth: none;<br>min_samples_split=2; min_samples_leaf=1;<br>min_weight_fraction_leaf=0; max_features: none;<br>max_leaf_nodes: none; min_impurity_split=0.0
RF<br>(random forest)	n_estimators=100; criterion: gini; max_depth: none;<br>min_samples_split=2; min_samples_leaf=1;<br>min_weight_fraction_leaf=0; max_features: none;<br>max_leaf_nodes: none
LR<br>(logistic regression)	Penalty: l2; Solver: lbfgs; multi_class: auto;<br>class_weight: none
LDA<br>(linear discriminant analysis)	Solver: svd; Shrinkage: none; Priors: none
KNN<br>(K-nearest neighbor<br>classification)	n_neighbors=5; weights: uniform; algorithm: auto;<br>n_jobs: none; p: none
